# Methionine Restriction Impairs Degradation of a Protein that Aberrantly Engages the Endoplasmic Reticulum Translocon

**DOI:** 10.17912/micropub.biology.001021

**Published:** 2023-11-09

**Authors:** Avery M. Runnebohm, Christopher J. Indovina, Samantha M. Turk, Connor G. Bailey, Cade J. Orchard, Lauren Wade, Danielle L. Overton, Brian J. Snow, Eric M. Rubenstein

**Affiliations:** 1 Department of Biology, Ball State University, Muncie, Indiana, United States; 2 Department of Biochemistry and Molecular Biology, Indiana University School of Medicine, Indianapolis, Indiana, United States; 3 St. Jude Graduate School of Biomedical Science, Memphis, Tennessee, United States; 4 AllSource PPS, United States; 5 Department of Geology, University of Georgia, Athens, Georgia, United States; 6 Flow Cytometry Department, LabCorp, United States; 7 Department of Biology, Indiana University – Purdue University Indianapolis, Indianapolis, Indiana, United States; 8 Department of Pathology and Laboratory Medicine, University of Wisconsin–Madison, Madison, Wisconsin, United States

## Abstract

Proteins that persistently engage endoplasmic reticulum (ER) translocons are degraded by multiple translocon quality control (TQC) mechanisms. In *Saccharomyces cerevisiae* , the model translocon-associated protein *Deg1* -Sec62 is subject to ER-associated degradation (ERAD) by the Hrd1 ubiquitin ligase and, to a lesser extent, proteolysis mediated by the Ste24 protease. In a recent screen, we identified nine methionine-biosynthetic genes as candidate TQC regulators. Here, we found methionine restriction impairs Hrd1-independent *Deg1* -Sec62 degradation. Beyond revealing methionine as a novel regulator of TQC, our results urge caution when working with laboratory yeast strains with auxotrophic mutations, often presumed not to influence cellular processes under investigation.

**Figure 1. Methionine restriction impairs Hrd1-independent degradation of Deg1 -Sec62:**
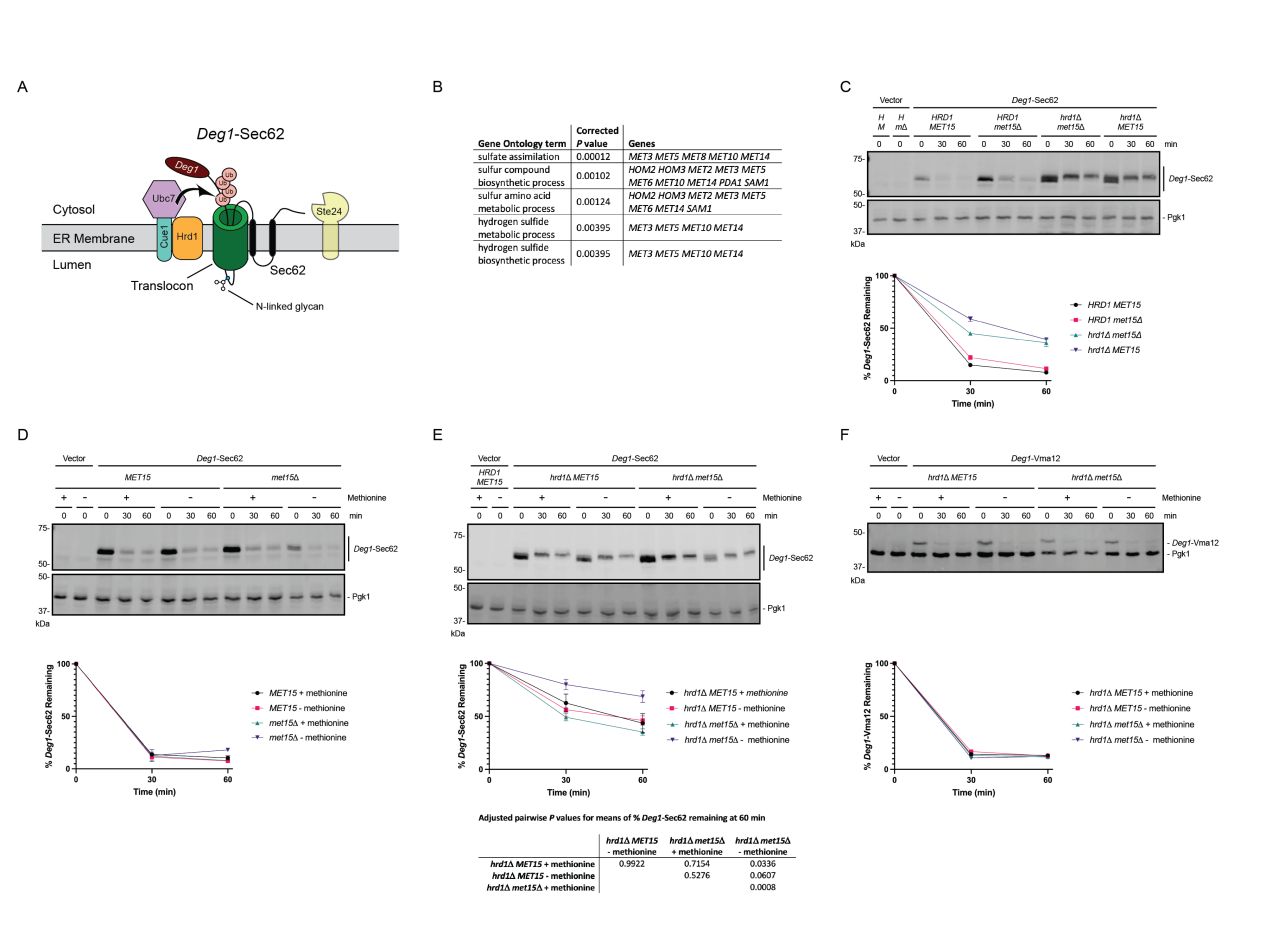
**(A)** Following persistent aberrant translocon engagement, *Deg1* -Sec62 degradation is promoted by the Hrd1 ubiquitin ligase (in concert with the Ubc7 ubiquitin-conjugating enzyme and its membrane anchor Cue1) and, to a lesser extent, by the zinc metalloprotease Ste24 and at least one additional mechanism (Runnebohm *et al.* 2020). *Deg1* -Sec62 undergoes N-linked glycosylation after aberrant translocon engagement (Rubenstein *et al.* 2012). Ub, ubiquitin. **(B)** Gene Ontology (GO) Process Terms (and associated genes) enriched in screen for requirements for *Deg1* -Sec62 degradation are listed. GO Terms were identified with the Gene Ontology Term Finder at the *Saccharomyces* Genome Database (www.yeastgenome.org/goTermFinder) using a *P* value cutoff of 0.01. Adapted from (Turk *et al.* 2023). **(C)** Cycloheximide chase of yeast of the indicated genotypes expressing *Deg1* -Sec62 cultured in synthetic defined (SD) medium containing methionine. *H* , *HRD1* . *M* , *MET15. m* Δ *, met15* Δ. **(D, E, F)** Yeast of the indicated genotypes expressing *Deg1* -Sec62 or *Deg1* -Vma12 were cultured to exponential phase in SD medium containing methionine, followed by a shift to fresh SD medium containing or lacking methionine, as indicated, for two hours before cycloheximide chase. For **(C)** and **(D)** , means of percent *Deg1* -Sec62 remaining for two biological replicates are plotted. Error bars indicating range are included, except for when the error bar would be shorter than the symbol. For **(E)** , means of percent *Deg1* -Sec62 remaining for three ( *hrd1* Δ *MET15* ) or six ( *hrd1* Δ *met15* Δ) biological replicates are plotted. Error bars indicate standard error of the mean. Means of percent *Deg1* -Sec62 remaining at 60 minutes in **(E)** were evaluated by one-way ANOVA ( *F* , 9.290; *P* , 0.0012) followed by Tukey’s multiple comparison test; adjusted pairwise *P* values are included beneath the plot. Pgk1, loading control. For **(F)** , values for one experiment are plotted.

## Description

Many endomembrane system and secreted proteins cross the eukaryotic endoplasmic reticulum (ER) membrane by passing through protein-conducting translocon channels. Proteins that persistently engage or clog translocons are targeted for degradation by one of several conserved translocon quality control (TQC) mechanisms (reviewed in (Wang and Ye 2020) ). Unresolved translocon clogging may cause or exacerbate metabolic and neurological disease (Colin *et al.* 2016; Muona *et al.* 2016; Kayatekin *et al.* 2018). The model engineered *Deg1* -Sec62 protein aberrantly engages translocons in *Saccharomyces cerevisiae* when its N-terminal tail aberrantly loops post-translationally into translocons and forms a stabilizing disulfide bond with the translocon interior (Rubenstein *et al.* 2012). *Deg1* -Sec62 is targeted for degradation by the ER-associated degradation (ERAD) ubiquitin ligase Hrd1 and, to a lesser extent, the zinc metalloprotease Ste24 and at least one uncharacterized mechanism ( Figure 1A ) (Rubenstein *et al.* 2012; Runnebohm *et al.* 2020).

To identify novel genes required for *Deg1* -Sec62 degradation, we recently conducted and reported a genome-wide, yeast growth-based reporter screen (Turk *et al.* 2023). Characterization of a subset of genes identified in this screen demonstrated that ERAD is broadly sensitive to perturbed lipid biosynthesis. Further, we found *Deg1* -Sec62 degradation is impaired by loss of the microtubule motor protein, Kar3, and, to a modest extent, of the histone methyltransferase Set2. Gene Ontology (GO) analysis ( www.yeastgenome.org ) of 128 candidate TQC-regulating genes identified in the screen revealed significant enrichment of genes linked to five processes associated with sulfur metabolism ( Figure 1B ). No additional Function, Component, or Process GO terms were enriched. Nine of the eleven genes in these categories encode enzymes in the methionine biosynthesis pathway.

Here, we tested the hypothesis that methionine is required for efficient *Deg1* -Sec62 degradation. We conducted cycloheximide chase experiments (Buchanan *et al.* 2016) to compare degradation of the steady state population of *Deg1* -Sec62 in yeast with wild type and deletion alleles of *HRD1* and *MET15* in Synthetic Defined (SD) media containing 0.001% methionine. *MET15* encodes O-acetyl homoserine-O-acetyl serine sulfhydrylase, which catalyzes synthesis of homocysteine, an immediate precursor of methionine. *MET15* is an auxotrophic marker gene deleted in several commonly used laboratory strains of *S. cerevisiae* (Brachmann *et al.* 1998). As previously observed (Engle *et al.* 2017; Runnebohm *et al.* 2020; Turk *et al.* 2023), loss of *HRD1* slowed *Deg1* -Sec62 degradation ( Figure 1C ). By contrast, *MET15* deletion did not impair degradation or exacerbate the degradation defect of yeast lacking *HRD1* under these conditions.

We considered the possibility that methionine in the growth medium suppresses an effect of *MET15* deletion and assessed *Deg1* -Sec62 degradation in *MET15* and *met15* Δ yeast in the context of methionine restriction. Yeast cultured to exponential-phase growth in medium containing methionine were shifted to fresh medium containing or lacking methionine for two hours ( Figure 1D ) prior to cycloheximide chase analysis. The rate of *Deg1* -Sec62 turnover was not altered regardless of the presence or absence of the *MET15* gene or of methionine in the medium.

We hypothesized methionine availability impacts Hrd1-independent TQC. Given the major role of Hrd1 in TQC, an effect of methionine on Hrd1-independent degradation may be masked by the presence and activity of Hrd1. Consistent with this, we observed that, despite reduced steady state abundance, two hours of methionine restriction significantly stabilized *Deg1* -Sec62 in *hrd1* Δ *met15* Δ yeast (but not *hrd1* Δ *MET15* yeast) ( Figure 1E ). After one hour, 69% of *Deg1* -Sec62 remained in methionine-restricted *hrd1* Δ *met15* Δ yeast, compared to 35% in *hrd1* Δ *met15* Δ yeast in media containing methionine. Thus, methionine restriction impairs at least one Hrd1-independent TQC degradative mechanism that acts on *Deg1* -Sec62.

Methionine restriction does not broadly impair ER protein quality control or the ubiquitin-proteasome system. First, Hrd1-dependent degradation of *Deg1* -Sec62 appears to be unaffected by methionine abundance ( Figure 1D ). Further, turnover of *Deg1* -Vma12, a transmembrane substrate of the Doa10 ERAD ubiquitin ligase (Ravid *et al.* 2006), proceeds unimpeded in methionine-restricted *hrd1* Δ *met15* Δ yeast ( Figure 1F ).

Several potential mechanisms may explain how methionine restriction impairs TQC degradation of *Deg1* -Sec62. We have previously shown that *STE24* deletion stabilizes and reduces steady state abundance of *Deg1* -Sec62 to a similar extent as methionine-restricted *hrd1* Δ *met15* Δ yeast (Runnebohm *et al.* 2020). This observation is consistent with methionine restriction and *STE24* deletion stabilizing *Deg1* -Sec62 via a common mechanism. Ste24 cleaves both prenylated CAAX box-containing proteins and non-prenylated proteins (Hildebrandt *et al.* 2016). It is unknown whether Ste24 directly cleaves *Deg1* -Sec62, which does not have a CAAX box. Maturation of cleaved, prenylated CAAX box substrates includes methylation of the newly exposed C-terminus, using the methyl donor S-adenosylmethionine, for which methionine is a direct precursor (Cherest and Surdin-Kerjan 1978; Hrycyna *et al.* 1991). Methionine depletion might disrupt maturation of a prenylated Ste24 substrate that promotes TQC degradation of *Deg1* -Sec62. Alternatively, methylation of translocon-associated proteins such as *Deg1* -Sec62 may facilitate direct recognition by Ste24 or other as-yet uncharacterized TQC machinery. Indeed, mass spectrometry analysis demonstrated methylation of two lysine residues within *Deg1* -Sec62 (see Supplemental Information in (Engle *et al.* 2017)). Future experiments will assess the requirement of *Deg1* -Sec62 methylation for its TQC degradation.

The effect of methionine restriction on TQC could be multi-faceted or indirect. The yeast response to methionine restriction is mediated in large part by the Skp-Cullin-F Box (SCF) ubiquitin ligase complex SCF ^Met30^ (reviewed in (Lauinger and Kaiser 2021) ). A shift in SCF ^Met30^ substrate specificity during methionine stress results in transcriptional reprogramming and cell cycle arrest. Our genome-wide screen revealed a modest *Deg1* -Sec62 degradation defect in yeast lacking *SET2* , which encodes a histone methyltransferase (Strahl *et al.* 2002; Turk *et al.* 2023). Further, loss of phospholipid methyltransferases Cho2 or Opi3 partially stabilizes *Deg1* -Sec62 (Henry *et al.* 2012; Turk *et al.* 2023). Thus, depletion of methionine and, by extension of S-adenosylmethionine, may impair degradation by altering SCF ^Met30^ substrate specificity, gene expression patterns, or membrane lipid composition. In any case, the impacts of dampened methylation on degradation would need to occur on the relatively short timescale of two hours (the period of methionine restriction in our experiments).

Despite impaired degradation, steady state abundance of *Deg1* -Sec62 was reduced in *hrd1* Δ *met15* Δ yeast following methionine restriction ( Figure 1E ), consistent with a diminished rate of synthesis. A similar reduction in *Deg1* -Vma12 abundance was not observed under these conditions ( Figure 1F ), even though expression of genes encoding both protein quality control substrates were driven by the *GPD* promoter. Further investigation will be required to understand how methionine restriction preferentially impacts steady state abundance of this TQC substrate. Unaltered *Deg1* -Vma12 degradation kinetics in methionine-restricted *hrd1* Δ *met15* Δ yeast indicates that altered *Deg1* -Sec62 abundance and degradation are not caused by broadly impaired ubiquitin-proteasome system or reduction in cell viability.

Combined with other recent studies (Oss *et al.* 2022), our results suggest caution is warranted when working with auxotrophic laboratory yeast strains, including those with *MET15* mutations. Auxotrophic mutations are often presumed to have negligible influence on cellular processes under investigation. Our experiments provide a counterexample. In the present case, the impact of impaired methionine synthesis by *MET15* deletion was mitigated by the presence of methionine in yeast growth medium. Given that many microbial experiments are conducted in growth medium containing low concentrations of nutrients whose synthesis may be impaired by auxotrophic gene mutation, this may not always be the case. Several genes encoding enzymes in the methionine biosynthesis pathway were identified in our yeast growth-based screen for requirements of *Deg1* -Sec62 degradation (Turk *et al.* 2023), suggesting that methionine may have become sufficiently depleted during the screen to allow for observable changes in growth in these mutants. Future experiments may directly test the requirement of other methionine and S-adenosylmethionine biosynthetic genes for TQC.

A failure to unclog stalled translocons reduces cellular health and may contribute to human disease (Ast *et al.* 2016; Colin *et al.* 2016; Muona *et al.* 2016; Kayatekin *et al.* 2018; Runnebohm *et al.* 2020). Cells have developed multiple partially overlapping mechanisms to resolve clogged translocons, reflecting the critical nature of protein movement across biological membranes. We reported here that methionine restriction impedes Hrd1-independent TQC. Methionine is an essential amino acid in humans. Deficiency or imbalance of methionine contributes to several chronic liver diseases (reviewed in (Li *et al.* 2020)). Our results suggest patients suffering from such conditions may experience disrupted hepatic TQC. A better understanding of the relationship between methionine physiology and degradation of translocon-clogging proteins could lead to improved therapeutic strategies for these maladies.

## Methods

**Yeast and Plasmid Methods.** Yeast were cultured at 30°C in Synthetic Defined (SD) growth medium lacking uracil (2% dextrose, 0.67% yeast nitrogen base, 0.002% adenine, 0.002% arginine, 0.001% histidine, 0.006% isoleucine, 0.006% leucine, 0.004% lysine, 0.001% methionine, 0.006% phenylalanine, 0.005% threonine, and 0.004% tryptophan; all percentages are w/v). For methionine restriction, methionine was omitted from the growth medium. To generate a plasmid encoding *Deg1* -Sec62 under the control of the *GPD* promoter, a 1.6-kB BamHI/HindIII fragment from pVJ30 (alias pRS414- *P _MET25_* - *Deg1* -Sec62) (Mayer *et al.* 1998) containing *Deg1* -Sec62 was inserted into the BamHI/HindIII sites of pVJ72 (alias p416GPD) (Mumberg *et al.* 1995). To generate a plasmid encoding *Deg1* -Vma12 under the control of the *GPD* promoter, a 1.5-kB BamHI/HindIII fragment from pVJ172 (alias pRS414- *P _MET25_* - *Deg1* -Vma12) (Ravid *et al.* 2006) containing *Deg1* -Vma12 was inserted into the BamHI/HindIII sites of pVJ72.

**Cycloheximide Chase, Cell Lysis, and Western Blotting.** Cycloheximide chase experiments were performed using exponential-phase cultures as described (Buchanan *et al.* 2016). For methionine restriction experiments, yeast were cultured to exponential growth in medium containing methionine. Two x 10 OD _600_ units (1 OD _600_ unit is 1 mL of yeast at an OD _600_ of 1.0) were harvested and centrifuged at 7000 x *g* for 5 min at room temperature. One cell pellet from each pair was washed three times with and resuspended in fresh medium with methionine. The other cell pellet was washed three times with and resuspended in fresh medium lacking methionine. Cultures were incubated for 2 hours at 30°C prior to cycloheximide chase. The presence or absence of methionine was maintained throughout the cycloheximide chase experiment. Protein extracts were prepared and analyzed by western blotting as described (Kushnirov 2000; Watts *et al.* 2015). At their C-termini, *Deg1* -Sec62 and *Deg1* -Vma12 possess two copies of the *Staphylococcus aureus* Protein A tag, which binds to mammalian immunoglobulins (Hjelm *et al.* 1972); thus, *Deg1* -Sec62 and *Deg1* -Vma12 were directly detected with AlexaFluor-680-conjugated rabbit-anti-mouse antibodies (Life Technologies, Inc.; 1:40,000). Pgk1 was detected with mouse anti-phosphoglycerate kinase 1 antibodies (clone 22C5D8; Life Technologies, Inc.; 1:20,000), followed by AlexaFluor-680-conjugated rabbit-anti-mouse antibodies (1:40,000). An Odyssey CLx Infrared Imaging System and Empiria Studio Software (Li-Cor) were used to image and analyze membranes. The ( *Deg1* -Sec62 or *Deg1* -Vma12)/Pgk1 signal intensity ratio was used to normalize protein abundance in a given experiment. Percent remaining was determined by comparing the ( *Deg1* -Sec62 or *Deg1* -Vma12)/Pgk1 signal intensity ratio at each time point to this ratio at 0 minutes for each culture.

**Statistical Analysis.** Data were analyzed using GraphPad Prism (version 10). Statistical analyses are described in the figure legend.

## Reagents

**Yeast strains used in this study.**

**Table:** 

**Name**	**Genotype**	**Source or Reference**
VJY22	*MATa his3* Δ *1 leu2* Δ *0 met15* Δ *0 ura3* Δ *0 hrd1* Δ:: *kanMX4*	(Tong *et al.* 2001)
VJY33	*MATa his3* Δ *1 leu2* Δ *0 ura3* Δ *0*	Gift of Mark Hochstrasser
VJY417	*MATa his3* Δ *1 leu2* Δ *0 met15* Δ *0 ura3* Δ *0*	Gift of Robert J. Tomko, Jr.
VJY478	*MATα his3* Δ *1 leu2* Δ *0 lys2* Δ *0 ura3* Δ *0 hrd1* Δ *::kanMX4*	Purchased from DharmaCon (YSC6272-201918569)

**Plasmids used in this study.**

**Table:** 

**Name**	**Alias**	**Description**	**Source or Reference**
pVJ27	pRS316	Empty vector (CEN, *URA3* , *AmpR* )	(Sikorski and Hieter 1989)
pVJ340	p416GPD- *Deg1* -Sec62	Plasmid (CEN, *URA3* , *AmpR* ) expressing *Deg1-* Sec62 driven by the *TDH3* ( *GPD* ) promoter. *Deg1* -Sec62 is a fusion protein consisting of, in sequence, *Deg1* (the N-terminal 67 amino acids from the yeast transcriptional repressor MATa2), a Flag epitope, Sec62, and two copies of a *Staphylococcus aureus* Protein A tag.	This study
pVJ343	p416GPD- *Deg1* -Vma12	Plasmid (CEN, *URA3* , *AmpR* ) expressing *Deg1-* Vma12 driven by the *TDH3* ( *GPD* ) promoter. *Deg1* -Vma12 is a fusion protein consisting of, in sequence, *Deg1* (the N-terminal 67 amino acids from the yeast transcriptional repressor MATa2), a Flag epitope, Vma12, and two copies of a *Staphylococcus aureus* Protein A tag.	This study
